# Atrial cardiomyopathy and incident ischemic stroke risk: a systematic review and meta-analysis

**DOI:** 10.1007/s00415-023-11693-3

**Published:** 2023-04-04

**Authors:** Jiahuan Guo, Dandan Wang, Jiaokun Jia, Jia Zhang, Fei Peng, Jingjing Lu, Xingquan Zhao, Yanfang Liu

**Affiliations:** 1 Department of Neurology, Beijing Tiantan Hospital, Capital Medical University, Fanyang Street 119, Beijing, 100070 China; 2 China National Clinical Research Center for Neurological Diseases, Beijing Tiantan Hospital, Capital Medical University, Beijing, China; 3 Research Unit of Artificial Intelligence in Cerebrovascular Disease, Chinese Academy of Medical Sciences, Beijing, China; 4 Center of Stroke, Beijing Institute for Brain Disorders, Beijing, China

**Keywords:** Atrial cardiomyopathy, Ischemic stroke, Markers, Meta-analysis

## Abstract

**Background and purpose:**

Growing evidence suggests that atrial cardiomyopathy may play an essential role in thrombosis and ischemic stroke. The aim of this systematic review and meta-analysis was to quantify the values of cardiomyopathy markers for predicting ischemic stroke risk.

**Methods:**

PubMed, Embase, and the Cochrane Library were searched for longitudinal cohort studies evaluating the association between cardiomyopathy markers and incident ischemic stroke risk.

**Results:**

We included 25 cohort studies examining electrocardiographic, structural, functional, and serum biomarkers of atrial cardiomyopathy involving 262,504 individuals. P-terminal force in the precordial lead V1 (PTFV1) was found to be an independent predictor of ischemic stroke as both a categorical variable (HR 1.29, CI 1.06–1.57) and a continuous variable (HR 1.14, CI 1.00–1.30). Increased maximum P-wave area (HR 1.14, CI 1.06–1.21) and mean P-wave area (HR 1.12, CI 1.04–1.21) were also associated with an increased risk of ischemic stroke. Left atrial (LA) diameter was independently associated with ischemic stroke as both a categorical variable (HR 1.39, CI 1.06–1.82) and a continuous variable (HR 1.20, CI 1.06–1.35). LA reservoir strain independently predicted the risk of incident ischemic stroke (HR 0.88, CI 0.84–0.93). N-terminal pro-brain natriuretic peptide (NT-proBNP) was also associated with incident ischemic stroke risk, both as a categorical variable (HR 2.37, CI 1.61–3.50) and continuous variable (HR 1.42, CI 1.19–1.70).

**Conclusion:**

Atrial cardiomyopathy markers, including electrocardiographic markers, serum markers, LA structural and functional markers, can be used to stratify the risk of incident ischemic stroke.

**Supplementary Information:**

The online version contains supplementary material available at 10.1007/s00415-023-11693-3.

## Introduction

Ischemic stroke is the second-leading cause of death and a major cause of disability worldwide [1]. Identifying stroke etiologies and early predictors of increased risk of ischemic stroke are of great significance. Standard evaluation fails to determine the etiology of one-third of ischemic stroke cases. There is evidence that most non-lacunar cryptogenic strokes are embolic and are known as embolic strokes of undetermined source (ESUS). Approximately one-sixth of patients remain ESUS after standard evaluation [2]. Initially, atrial fibrillation (AF) was considered the main cause of these ESUS cases. However, AF was difficult to detect since it can be occult and paroxysmal. The temporal relationship between AF and incident ischemic stroke was also not certain [3]. In addition, although the New Approach Rivaroxaban Inhibition of Factor Xa in a Global Trial versus ASA to Prevent ESUS (NAVIGATE) trial failed to show an overall benefit of anticoagulation [4], evidence demonstrated that anticoagulation was more effective than aspirin in a subset of patients with ESUS who are at greater risk of having AF [5]. Atrial cardiomyopathy has been proposed as an entity including the spectrum of structural, architectural, contractile or electrophysiological changes affecting the left atrium (LA), which may present before AF and worsen with the development of AF [6, 7]. Recently, several lines of evidence have indicated that AF may only be a secondary manifestation and an exacerbating factor for atrial cardiomyopathy [8, 9]. This has prompted increasing interest in elucidating atrial cardiomyopathy as a potential substrate of both AF and ischemic stroke. In patients with AF, more severe atrial cardiomyopathy is associated with an increased risk of ischemic stroke [10]. Moreover, emerging evidence suggests an association between abnormal atrial cardiomyopathy markers and an increased risk of ischemic stroke even in the absence of AF [11]. Taken together, an improved understanding of the role of atrial cardiomyopathy in ischemic stroke is of great significance for high-risk population identification, stroke prevention, etiology classification and treatment decision making. However, there is no uniform definition of atrial cardiomyopathy to date, a variety of markers have been used to define atrial cardiomyopathy in different studies [8]. In addition, the results from studies are inconsistent.

Accordingly, we aim to systematically review the current diagnostic biomarkers of atrial cardiomyopathy and further quantify their value for predicting ischemic stroke risk in the general population through meta-analyses.

## Methods

This systematic review and meta-analysis were conducted in accordance with consensus guidelines [12] and were reported according to the recommendations of the PRISMA 2020 statement. The study protocol was registered and published in the International Prospective Register of Systematic Reviews (PROSPERO: CRD42022353852).

### Search strategy

The PubMed, Embase, and Cochrane Library databases were searched from 01/01/2002 to 06/08/2022, without language restrictions. Two investigators (JHG and YFL) systematically and independently searched the databases using the search syntax for concepts of ischemic stroke and cardiomyopathy markers that are based on PubMed search syntax and modified accordingly for other search engines. The detailed search strategy is described in Supplementary Table 1.

### Eligibility criteria

According to the population, intervention, comparison, outcome and study design (PICOS) system, studies were eligible if they met the following criteria: (1) general population without stroke at baseline; (2) appropriate measurements of at least one marker of atrial cardiomyopathy. The markers were classified into three main categories, including electrocardiographic, imaging, and serum biomarkers (Supplementary Table 2). (3) The outcome was incident ischemic stroke. (4) Prospective or retrospective cohort studies estimated the risk for ischemic stroke with hazard ratios (HRs) and 95% CIs derived from a multivariate Cox regression analysis.

### Study selection

After removing duplicates, two independent reviewers (JHG and YFL) screened the titles and abstracts of all literature. The full texts of the potentially relevant articles were then retrieved to assess eligibility for inclusion by the two reviewers. Disagreements were resolved by discussion with a third reviewer (JZ) when necessary.

### Data extraction

A template spreadsheet was used to extract study details and results (author names, year of publication, country, study design, population characteristics, including data source, sample size, duration of follow-up, determination of outcomes, risk estimates and adjustments). Two investigators independently performed data extraction and quality evaluation.

### Risk of bias assessment

The study quality of the final included studies was evaluated according to the Newcastle‒Ottawa Scale (NOS) scoring from 0 to 9 according to three aspects: selection of the patients, comparability of cohorts and outcome evaluation. Studies with an NOS score higher than 6 were considered to be of high quality.

### Statistical analysis

Review Manager software version 5.4 was used for statistics and analysis. Risk estimates of HRs and their 95% confidence intervals (HRs) were used to evaluate the association between atrial cardiomyopathy markers and ischemic stroke. The cardiomyopathy markers were analyzed separately as categorical variables and continuous variables. When analyzed as categorical variables, the highest level of atrial cardiomyopathy markers was compared to the group with the lowest levels.

We used the I-squared (*I*^2^) statistic to assess the degree of study heterogeneity. An *I*^2^ value ≤ 25% indicates insignificant heterogeneity, 26–50% indicates low heterogeneity, 51% to 75% indicates moderate heterogeneity and ≥ 76% indicates high heterogeneity. The fixed-effects model was used when study heterogeneity was low. Otherwise, a random-effect model was used. Publication bias was evaluated by funnel plots. Sensitivity analysis was conducted (1) only including studies with patients free of clinical AF; (2) excluding studies that used ischemic stroke or TIA as an outcome; (3) including only studies at low risk of bias; and (4) omitting each study in turn.

## Results

### Search results

**Fig. 1:**
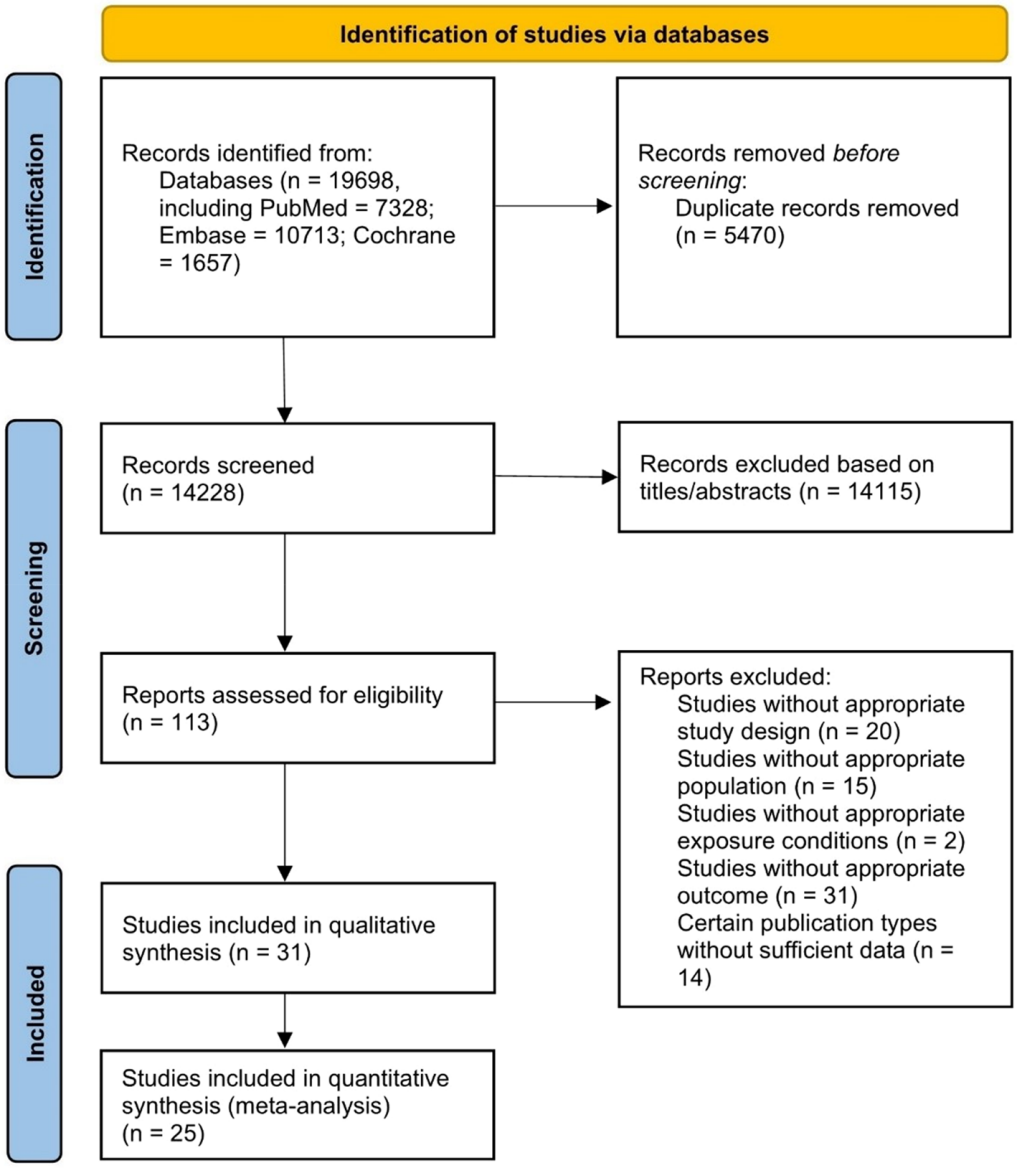
Flowchart of the study selection for the systematic review and meta-analysis

The flow chart demonstrates the selection of studies for this systematic review and meta-analysis (Fig. 1). We initially identified 19,698 potentially eligible articles (PubMed = 7328; Embase = 10,713; Cochrane Library = 1657). Among them, 5470 duplicate studies and 14,115 studies were excluded after screening titles and abstracts. We assessed 113 full texts for eligibility, 31 studies were identified that fulfilled the eligibility criteria and were included in the qualitative synthesis. Across these studies, a total of 25 cohort studies were used for the meta-analysis. Electrocardiographic markers were assessed in 7 studies, imaging markers were assessed in 12 studies, serum biomarkers were assessed in 9 studies.

### Characteristics of the studies

The basic characteristics of each included study are summarized in Supplementary Table 3. The 25 cohort studies used in the meta-analysis included 262,504 participants with a mean age between 52 and 86 years. A total of 10,297 ischemic stroke cases were identified. Among the cohort studies included in the meta-analyses, 1 study had an NOS score of 4, 1 study had an NOS score of 5 and 4 studies had an NOS score of 6, which raised concerns about selection and outcome bias. The remaining studies were all of high quality (Supplementary Table 4). Possible publication bias was assessed using funnel plots (Supplementary Figs. 1–3).

### Association between ECG parameters and incidence of ischemic stroke

#### PTFV1

**Fig. 2:**
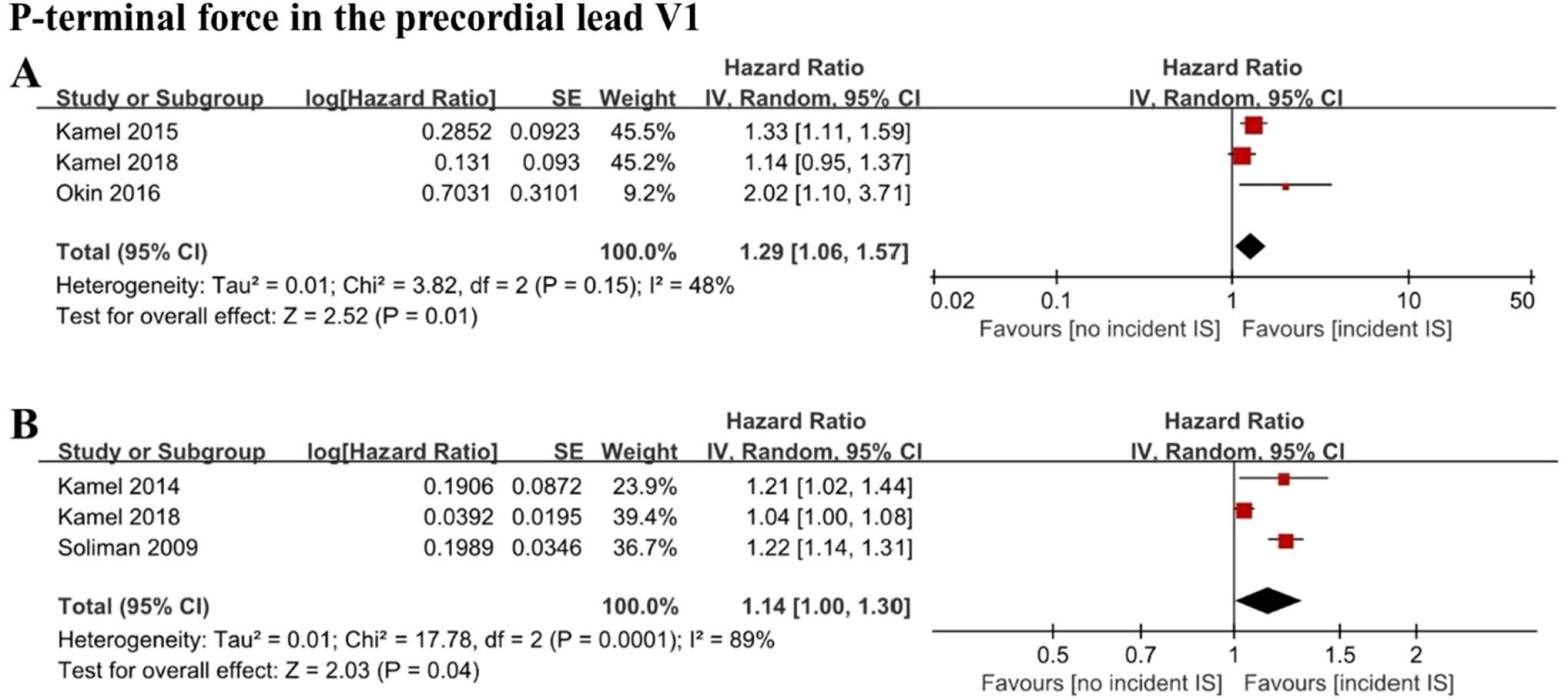
Forest plot demonstrating the value of PTFV1 in predicting ischemic stroke, with PTFV1 analyzed as a categorical variable (**A**) or continuous variable (**B**). PTFV1: P-terminal force in the precordial lead V1

When P-terminal force in the precordial lead V1 (PTFV1) was analyzed as a categorical variable [11, 13, 14], 3 cohort studies with 20,144 individuals were included. All participants were free of AF at baseline. Pooled results showed that individuals with PTFV1 higher than 4000 μV*ms had a higher risk of incident ischemic stroke (HR 1.29; 95% CI 1.06–1.57; *I*^2^ = 48%) (Fig. 2A). Sensitivity analyses showed consistent results. When PTFV1 was analyzed as a continuous variable [11, 15, 16], 3 cohort studies with 25,893 individuals were included. All participants were free of AF at baseline. Increased PTFV1 levels were also associated with incident ischemic stroke (HR 1.14; 95% CI 1.00–1.30; *I*^2^ = 89%) (Fig. 2B). Excluding one study that involved overadjustment [11] did not change our main results while reducing heterogeneity to 0.

#### Other P wave indices

**Fig. 3:**
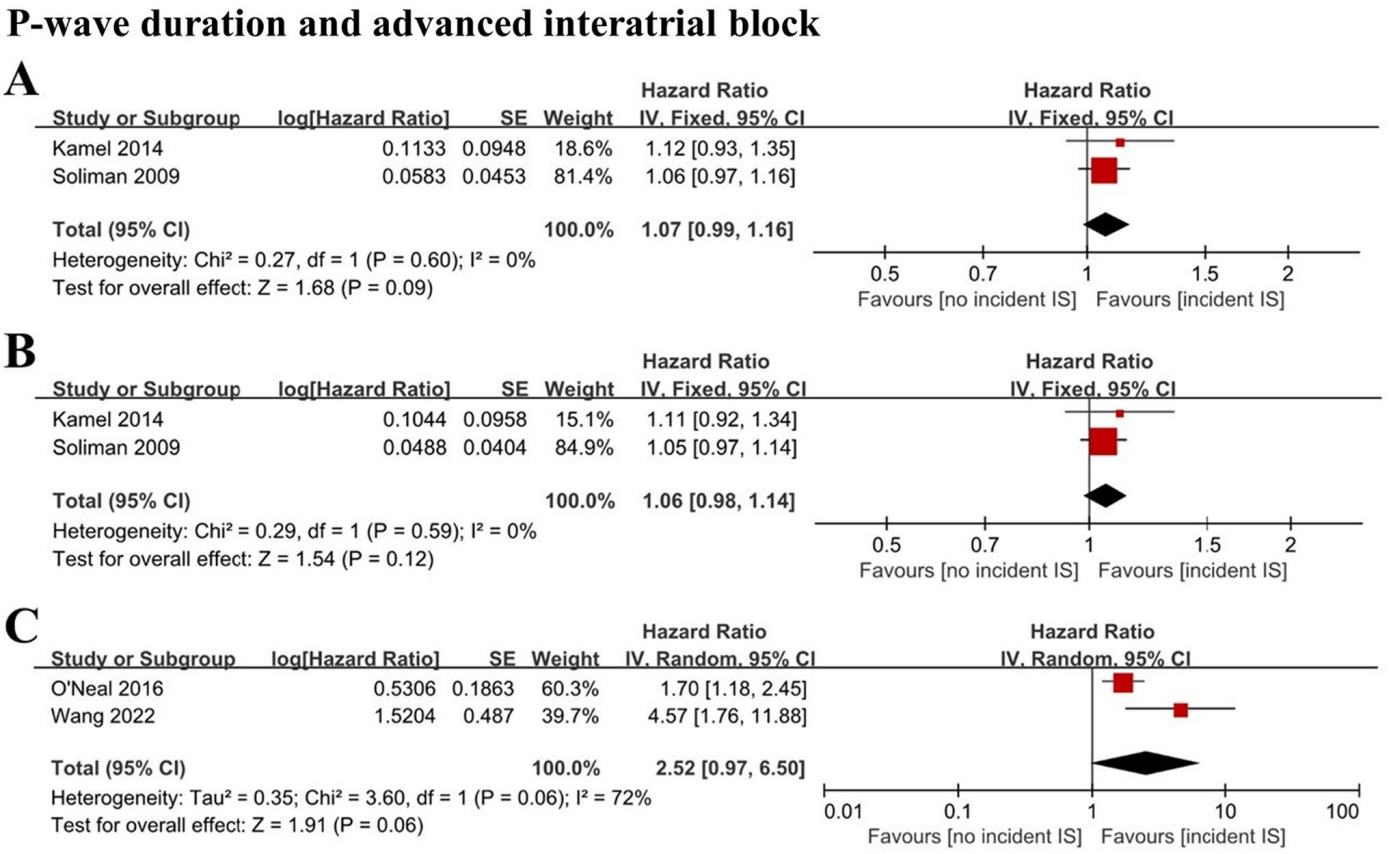
Forest plot demonstrating the value of maximum P-wave duration (**A**), mean P-wave duration (**B**) and advanced interatrial block (**C**) in predicting ischemic stroke

**Fig. 4:**
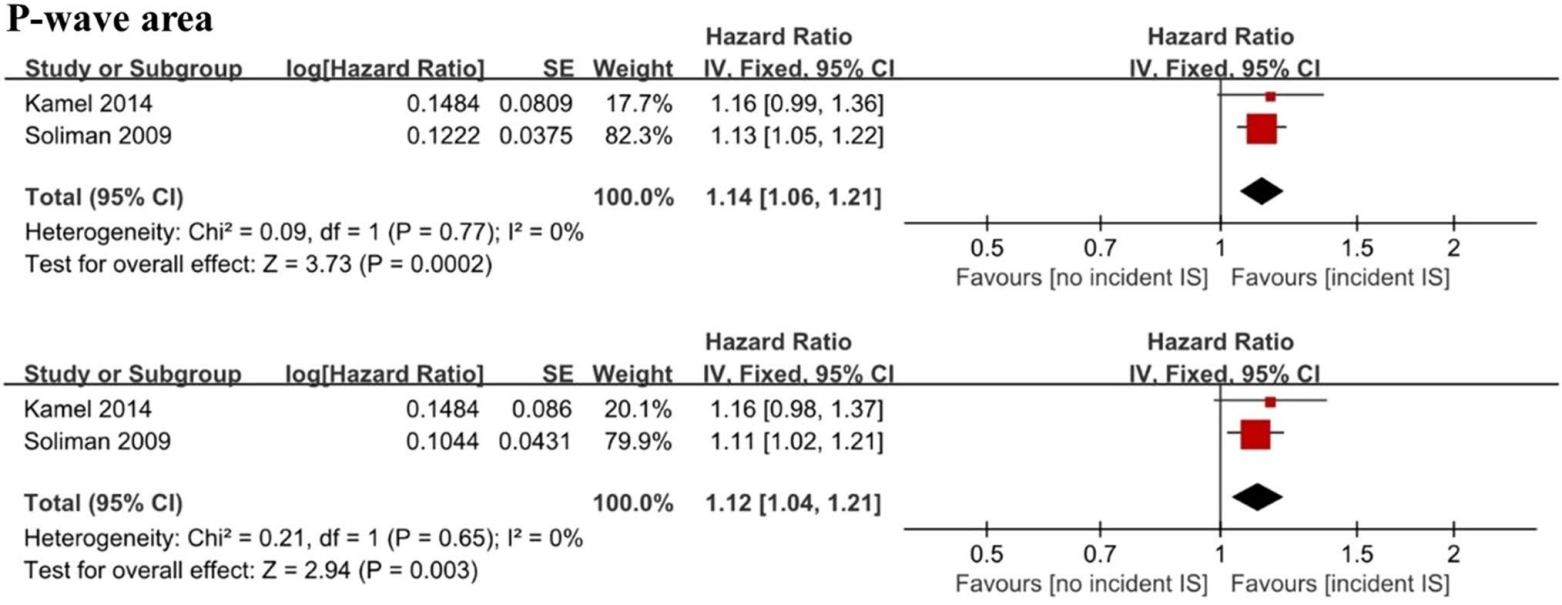
Forest plot demonstrating the value of the maximum P-wave area (**A**) and mean P-wave area (**B**) in predicting ischemic stroke

Two cohort studies with 22,710 individuals analyzed P-wave duration as a continuous variable [15, 16] and demonstrated that maximum P-wave duration (HR 1.07; 95% CI 0.99–1.16; *I*^2^ = 0%) (Fig. 3A) and mean P-wave duration (HR 1.06; 95% CI 0.98–1.14; *I*^2^ = 0%) (Fig. 3B) were not associated with incident ischemic stroke. When the maximum P-wave duration was higher than 120 ms, it was defined as advanced interatrial block (aIAB). Analysis of two cohort studies with 15,232 individuals [17, 18] showed that there was no association between aIAB and incident ischemic stroke (HR 2.52; 95% CI 0.97–6.50; *I*^2^ = 72%) (Fig. 3C). The pooled analysis of 2 studies [15, 16] demonstrated that the maximum P-wave area (HR 1.14; 95% CI 1.06–1.21; *I*^2^ = 0%) (Fig. 4A) and mean P-wave area (HR 1.12; 95% CI 1.04–1.21; *I*^2^ = 0%) (Fig. 4B) were associated with increased ischemic stroke risk.

### Association between echocardiographic biomarkers and the incidence of ischemic stroke

#### LA size

**Fig. 5:**
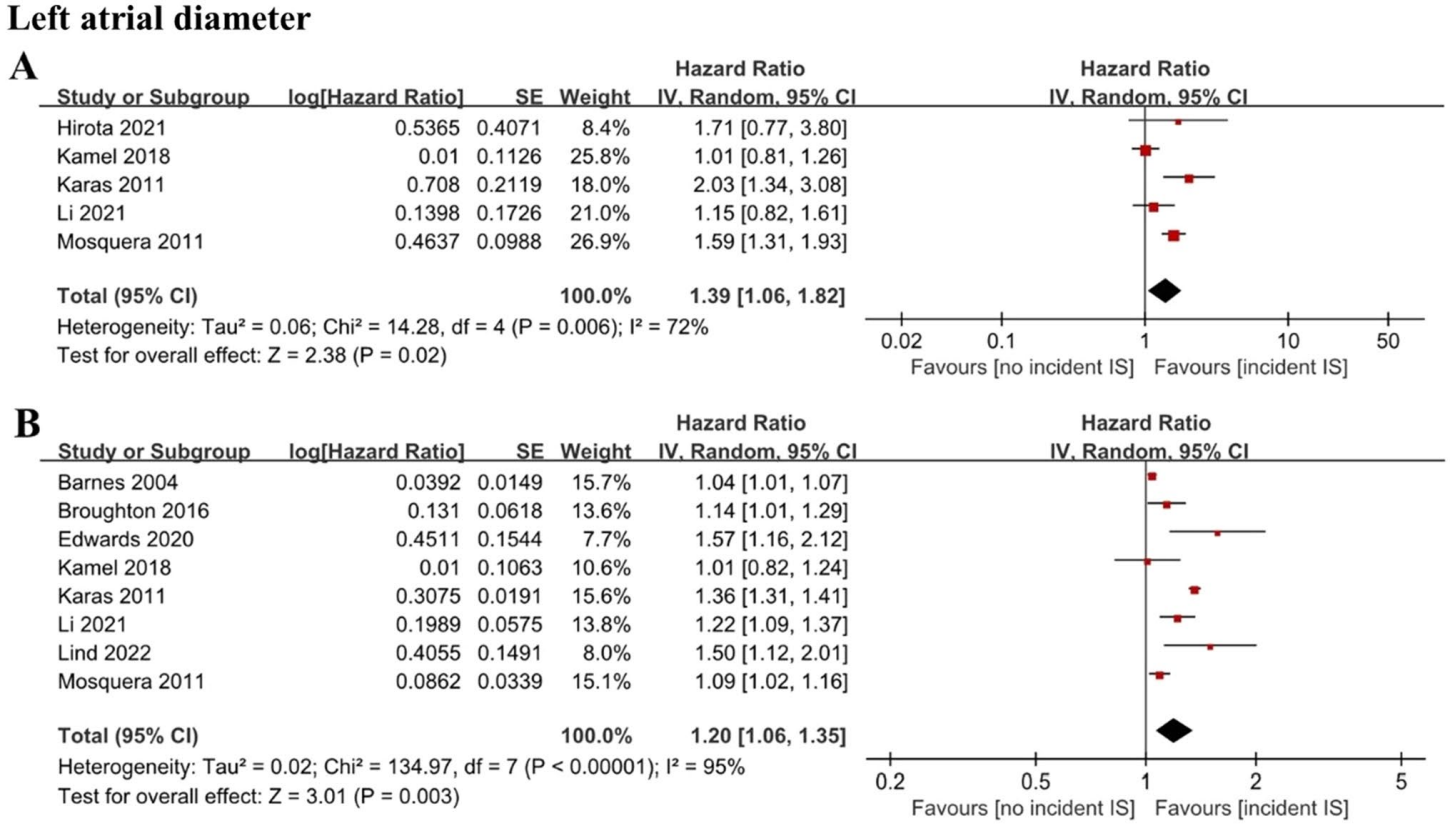
Forest plot demonstrating the value of LA diameter in predicting ischemic stroke, with LA diameter analyzed as a categorical variable (**A**) or continuous variable (**B**). *LA* left atrial

When analyzed as a categorical variable, the pooled results of five studies with 88,702 individuals [11, 19–22] showed that a higher LA diameter was related to an increased risk of ischemic stroke (HR 1.39; 95% CI 1.06–1.82; *I*^2^ = 72%) (Fig. 5A). When analyzed as a continuous variable, the pooled results of 8 cohort studies with 95,201 individuals [11, 19, 20, 22–26] also showed that LA diameter levels were associated with incident ischemic stroke (HR 1.20; 95% CI 1.06–1.35; *I*^2^ = 95%) (Fig. 5B). Sensitivity analyses included only enrolled high-quality studies (HR 1.15; 95% CI 1.05–1.25; *I*^2^ = 53%), and omitting each study in turn did not change the main results. However, analysis of studies that only included patients free of prior AF and AF at baseline demonstrated no significant association between LA diameter and increased risk of ischemic stroke (HR 1.13; 95% CI 0.93–1.37; *I*^2^ = 72%).

#### LA function

**Fig. 6:**
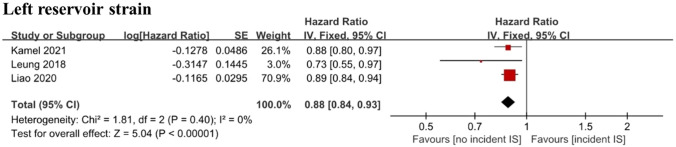
Forest plot demonstrating the value of LA reservoir strain in predicting ischemic stroke. *LA* left atrial

Three cohort studies with 11,079 individuals [27–29] investigated the association between LA reservoir strain and the risk of ischemic stroke. The pooled results demonstrated an inverse association between LA reservoir strain and ischemic stroke risk (HR 0.88; 95% CI 0.84–0.93; *I*^2^ = 0%) (Fig. 6). Sensitivity analyses showed consistent results.

### Association between serum biomarkers and the incidence of ischemic stroke

#### NT-proBNP

**Fig. 7:**
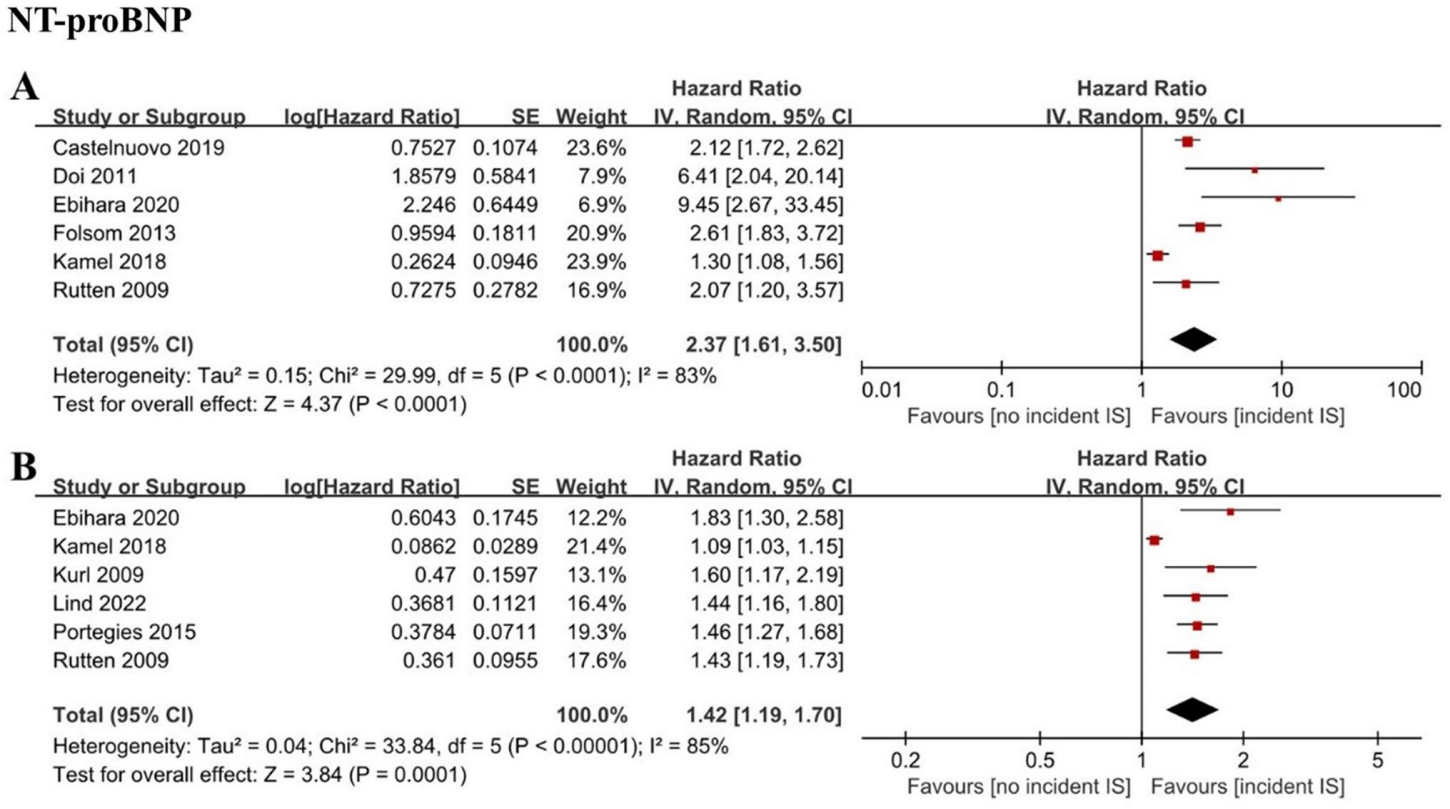
Forest plot demonstrating the value of NT-proBNP in predicting ischemic stroke, with NT-proBNP analyzed as a categorical variable (**A**) or continuous variable (**B**). *NT-proBNP* N-terminal pro-brain natriuretic peptide

When N-terminal pro-brain natriuretic peptide (NT-proBNP) was analyzed as a categorical variable, 6 cohort studies of high quality with a total of 85,358 individuals were included in the meta-analysis [11, 30–34]. The pooled results showed that a higher level of NT-proBNP was associated with an increased risk of ischemic stroke in the highest vs. lowest analyses (HR 2.37; 95% CI 1.61–3.50; *I*^2^ = 83%) (Fig. 7A). Sensitivity analysis by deleting one study in turn did not change the result. Sensitivity analysis of patients free of prior AF was not performed since only one study reported these data. Six cohort studies with 20,711 individuals analyzed NT-proBNP as a continuous variable [11, 26, 30, 34–36]. A significant association was observed between NT-proBNP levels and incident ischemic stroke risk (HR 1.42; 95% CI 1.19–1.70; *I*^2^ = 85%) (Fig. 7B). Excluding one study that involved overadjustment [11] did not change our main results while reducing heterogeneity to 0. Other sensitivity analyses showed consistent results.

## Discussion

To our knowledge, this is the first meta-analysis that reports the association between current diagnostic biomarkers of atrial cardiomyopathy and ischemic stroke risk. Pooling data from 25 cohort studies demonstrated that atrial cardiomyopathy markers, including electrophysiological markers (PTFV1, P-wave area), LA size parameters (LA diameter), LA function parameters (LA reservoir strain) and serum biomarkers (NT-proBNP), were all significantly associated with increased ischemic stroke risk. Our results highlight that atrial cardiomyopathy markers are promising for risk stratification and prevention of ischemic stroke. In addition, abnormal biomarkers of atrial cardiomyopathy may be highly suggestive of cardioembolic etiology of cryptogenic stroke.

In terms of atrial electrophysiology, several P-wave parameters that reflect underlying atrial size, atrial structural and electrical activation have been studied. A previous meta-analysis conducted in 2017 demonstrated that PTFV1, P-wave area and P-wave duration are independently associated with incident ischemic stroke [37]. Our meta-analysis found a similar result in the relationship between PTFV1 and P-wave area and ischemic stroke, but the results in our meta-analysis indicated no significant association between P-wave duration and ischemic stroke risk. Several reasons might contribute to this discrepancy. First, the former meta-analysis included both cross-sectional and cohort studies, which resulted in obvious heterogeneity. Our study, on the other hand, only included cohort studies in the general population. Second, the previous study was conducted 5 years ago, and several new studies were published during this period. In general, our study updated the results and provided more reliable evidence on the relationship between P-wave parameters and ischemic stroke. In addition, an analysis conducted by Maheshwari et al. [38] showed that an abnormal P-wave axis (aPWA) was also independently associated with ischemic stroke. The same author further confirmed that considerations of the aPWA can improve the ischemic stroke predictive ability of the CHA2D2-VASc score. The PR interval, on the other hand, was not associated with incident ischemic stroke risk in a cohort conducted by Soliman et al. [15] However, these findings should be further confirmed due to the small numbers of studies.

LA enlargement, a hallmark of LA remodeling, may be detected before the onset of AF and is known as one of the markers of atrial cardiomyopathy [39]. Emerging data suggest that LA enlargement is independently associated with cardiovascular events [40]. A previous meta-analysis revealed a strong association between LA diameter and a combination of incident stroke and thromboembolic events [41]. Our meta-analysis updated the results and provided further evidence for the relationship between LA diameter and ischemic stroke as an independent outcome. Notably, although our study demonstrated a consistent result that LA diameter was associated with a greater risk of ischemic stroke, the significant association disappeared when we only included studies that enrolled individuals free of AF. This indicates that LA diameter may be a useful predictor for future ischemic stroke in AF patients but not in those without AF. It is of note that the heterogeneity of the results was relatively large. Therefore, these results should be interpreted with caution and more high-quality studies are needed in the future to confirm these results. Other than LA diameter, the LA volume index may be a more precise and suitable parameter of LA size. However, evidence from large perspective cohort studies on the relationship between LA volume index and ischemic stroke is limited and controversial. A cohort study by Barnes et al. demonstrated that a higher LA volume index increased the risk of ischemic stroke [23], while another study conducted by Habibi et al. indicated no statistical association [42]. Additionally, one study used LA diameter indexed by body surface area [43], and another study used LA diameter indexed by height [44] as LA size parameters, further confirming the association between LA size and ischemic stroke risk. We did not include these studies in the meta-analysis due to the lack of number (only one study for each index). The results should be interpreted with caution, and further studies are needed.

In addition to structural abnormalities, atrial function may provide further indicators of atrial cardiomyopathy. There was evidence suggesting that LA function has prognostic value for cardiovascular events that are over and above that provided by LA size [45]. We included three cohort studies in our meta-analysis, all demonstrating that higher LA strain was associated with a lower risk of ischemic stroke [27–29]. Our pooled result showed a 0.88-fold reduced ischemic stroke risk in participants with a higher LA strain. Consistently, Liao et al. demonstrated that LA systolic and early diastolic strain rates were also predictors of future ischemic stroke [28]. LA emptying fractions measured with cardia magnetic resonance, a marker of global/reservoir LA function, were reported to be inversely associated with ischemic stroke risk in a recent study [42]. The onset of P-wave to A’ duration on tissue Doppler imaging (PA-TDI) is a marker that reflects the total atrial activation time and is a surrogate for fibrosis and LA substrate remodeling. In a cohort study conducted by Leung, the PA-TDI was independently associated with ischemic stroke after correcting for traditional risk factors [27]. Since only one study targeted the general population free of prior AF [29], whether atrial function abnormalities are independent predictors for ischemic stroke in individuals free of AF needs to be further studied.

NT-proBNP is a cardiac hormone secreted by myocytes as a reaction to several stimuli, including cardiac wall stretch. NT-proBNP showed a strong correlation with echocardiographic parameters of LA remodeling and is a marker of atrial dysfunction [46]. Increased NT-proBNP has been associated with an increased risk of cardiovascular events and death [47]. Our pooled analysis further confirmed that higher levels of NT-proBNP were associated with increased ischemic stroke risk, even in patients free of AF. Notably, study heterogeneity was relatively large, publication bias may exist, especially in studies in which NT-pro BNP was analyzed as a continuous variable. After a study that may involve overadjustment was excluded in the sensitivity analysis, the heterogeneity was significantly reduced and the main results were not changed. In addition, it is plausible to assume that there may be an interaction between NT-proBNP and LA diameter since they are correlated. However, only a few studies provided both two parameters at the same time, thus it is not sufficient to support the analysis of interactions between the parameters. Future studies should consider including all these parameters to determine whether there are interactions between them. Other atrial cardiomyopathy markers, such as LA fibrosis, may also be reliable predictors of ischemic stroke. King et al. [48] used late gadolinium enhancement-cardiac magnetic resonance imaging to quantify LA fibrosis and demonstrated that more severe LA fibrosis is associated with an increased risk of ischemic stroke and TIA. However, it is difficult to use LA fibrosis for large-scale screening due to the particular methodology for LA fibrosis detection.

The exact mechanisms linking atrial cardiomyopathy and ischemic stroke are uncertain, several possible explanations can be envisaged. First, LA dysfunction may be a marker of the presence of occult AF or future AF episodes that lead to ischemic stroke. Second, atrial cardiomyopathy may be the primary driver of thrombus development in the LA due to blood stasis and a hypercoagulable state associated with atrial fibrosis, endothelial damage and inflammation, even in the absence of AF [42, 49, 50]. Overall, acknowledging atrial cardiomyopathy as a predictor of ischemic stroke is of great clinical significance. First, atrial cardiomyopathy markers can be easily detected, which makes them suitable for risk-stratified screening and the development of primary prevention strategies in the general population. Individuals with abnormal levels of atrial cardiomyopathy markers may benefit from more frequent rhythm monitoring to detect subclinical AF and inform earlier prevention. Second, atrial cardiomyopathy biomarkers may help identify probable cardioembolic ischemic stroke subtypes, especially in cryptogenic stroke patients without clinical overt AF. In addition, by refining a population with stroke and biomarkers of atrial cardiomyopathy, physicians can identify a patient most likely to benefit from anticoagulation therapy. Medical therapies for atrial cardiomyopathy and whether they can lower ischemic stroke risk in susceptible populations are of great clinical value and deserve further investigation.

## Limitations

Our systematic review and meta-analysis has several limitations. First, only observational studies were included. Despite the inclusion of cohort studies with multivariate analysis, residual confounding factors may exist, and causation cannot be proven. Second, markers of atrial cardiomyopathy were analyzed as categorical or continuous variables in different studies, and it was not possible to pool all the studies together. Third, we limited our research to studies published within the past 20 years to ensure that our results are applicable to current practice. Fourth, treatment information during the follow-up was not demonstrated in the cohort studies that were included in our article, which may contribute to some bias. Finally, there is a lack of information on ischemic stroke etiology in the studies included in our analysis. Future studies focused on the relationship between atrial cardiomyopathy and ischemic stroke risk may take stroke etiology into consideration.

## Conclusions

Electrical, structural, functional, and biochemical markers of atrial cardiomyopathy can be used to predict incident ischemic stroke. These markers are of great clinical value in stroke risk stratification in the general population. In addition, abnormal atrial cardiomyopathy markers may have implications for the potential cardioembolic etiology of cryptogenic stroke patients and for therapeutic interventions. Further studies are needed to validate the causal relationship between atrial cardiomyopathy and guide future prevention and therapeutic strategies.

## Supplementary Information

Below is the link to the electronic supplementary material.

Supplementary file1 (DOCX 332 KB)

## Data Availability

Some or all datasets generated during and/or analyzed during the current study are not publicly available but are available from the corresponding author on reasonable request.
