# Supplementary material for: Atrial cardiomyopathy and incident ischemic stroke risk: a systematic review and meta-analysis
Source: J Neurol. 2023 Apr 4;270(7):3391–401. doi: 10.1007/s00415-023-11693-3 (PMC10267254; doi:10.1007/s00415-023-11693-3)

**Supplementary Table 1.** Detailed description of the search strategy

| **PubMed** | |
| --- | --- |
| #1 | "Ischemic Stroke"[Mesh] |
| #2 | stroke [Title/Abstract] |
| #3 | Ischemi*[Title/Abstract]) OR ischaemi*[Title/Abstract] OR cryptogenic [Title/Abstract] OR wake up[Title/Abstract] OR wake-up[Title/Abstract] OR embolic[Title/Abstract] |
| #4 | atrial OR atrium OR LA |
| #5 | "Cardiomyopathies"[Mesh] |
| #6 | cardio* AND myopathy* |
| #7 | cardiomyopathy* OR myocardiopathy* OR enlargement OR volume OR size OR function* OR biomarker* OR marker* OR morphology* OR structure* OR remodel* OR electrocardio* OR echocardiog* OR diameter OR index OR fraction* OR strain OR low-voltage OR fibrosis |
| #8 | P-terminal force OR PTFV* OR Interatrial block OR P-wave OR P wave OR N-terminal pro-B-type natriuretic peptide OR N-terminal pro-BNP OR NTproBNP OR N-BNP peptide OR NT-BNP OR Amino-terminal pro-brain natriuretic peptide OR aminoterminal pro-B-type natriuretic peptide OR NT-proBNP OR Midregional pro-atrial natriuretic peptide OR MR-proANP |
| #9 | #2 AND #3 |
| #10 | #1 OR #9 |
| #11 | #5 or #6 or #7 |
| #12 | #4 AND #11 |
| #13 | #8 OR #12 |
| #14 | #10 AND #13 |

**Supplementary Table 2. Atrial cardiomyopathy markers included**

| Category | Markers | Method | Definition |
| --- | --- | --- | --- |
| Electrocardiographic markers | P-terminal force in the precordial lead V1 (PTFV1) | 12-lead ECG | The duration (ms) of the downward deflection (terminal portion) of the P wave in lead V1 multiplied by the absolute value of its amplitude |
|  | P-wave duration |  | The duration (ms) of P wave |
|  | interatrial block |  | P wave duration longer than 120 ms with or without biphasic morphology in leads II, III, and Avf |
|  | P-wave area |  | A summation of all P-wave amplitudes at respective sampling points multiplied by the sampling interval which were calculated by summing the absolute values of the areas of the upward and downward deflections of the P wave |
|  | Abnormal P-wave axis |  | Any P-wave axis value outside 0 to 75 °, which was determined by measuring the positive or negative P-wave deflections on all 6 limb leads and then calculating the net direction of electric activity using the hexaxial reference system |
|  | PR interval |  | P-wave duration (ms) plus the mean PR-segment duration (ms) |
| Structural markers | Left atrial (LA) diameter | Echocardiography | measured from the posterior aortic wall to the posterior left atrial wall, in the parasternal long-axis view at the end-ventricular systole |
|  | LA volume index |  | LA volume indexed to body surface area |
| Functional markers | LA reservoir strain | Two-dimensional speckle tracking echocardiography | A parameter represents the degree of deformation as the left atrium fills and stretches during ventricular systole |
|  | LA ejection fraction | Cardia magnetic resonance | A left function parameter calculated as (maximum LA volume-minimum LA volume)/ maximum LA volume *100% |
|  | P-wave to A’ duration on tissue Doppler imaging (PA-TDI) | Tissue Doppler imaging | The onset of P-wave to A’ duration on tissue Doppler imaging, representing the time duration between electrical and mechanical activation of the LA measured on colourcoded TDI, reflects the total atrial activation time |
| Serum biomarker | N-Terminal Pro-Brain Natriuretic Peptide (NT-proBNP) | Blood sample testing | A cardiac hormone secreted by myocytes as a reaction to several stimuli, including cardiac wall stretch |
| Myocardial fibrosis | Atrial fibrosis in MRI | Late gadolinium enhancement cardiac magnetic resonance imaging | The quantity of late gadolinium enhancement (LGE) observed on cardiac magnetic resonance |

| **Supplementary Table 3.** Basic characteristics of the articles included in the meta-analysis | | | | | | | | | | |
| --- | --- | --- | --- | --- | --- | --- | --- | --- | --- | --- |
| **First author, year** | **Country** | | **Population** | **Atrial cardiomyopathy marker(s)** | **Sample sizes (total/stroke event)** | **Mean age/Male (%)** | **History of**  **Hypertension**  **/Diabetes (%)** | **Ischemic stroke**  **diagnosis** | **Follow-up period** | **Qualityscore** |
| Barnes,  2004 | UK | | ≥65 yrs free of stroke, TIA and AF | LA diameter,LA volume, LA volume  index | 1554/92 | 75.1/41 | 55/10 | Medical records | 4.3 years | 6 |
| Broughton,  2016 | USA | | The Cardiovascular Health Study, ≥65 yrs | LA diameter | 4572/739 | No  specific | -/15 | Medical records | 13 years | 7 |
| Castelnuovo,  2019 | Italy | | The harmonized database and biobank of the Biomarkers for Cardiovascular Risk Assessment in Europe, free of stroke | NT-proBNP | 58173/1176 | 52/50 | -/5 | ICD-8, ICD-9  and ICD-10  codes | 7.9 years | 7 |
| Doi,  2011 | Japan | | ≥40 yrs, free of cardiovascular disease | NT-proBNP | 3104/54 | 61.3/42 | 42/17 | ICD-10 codes | 5 years | 7 |
| Ebihara,  2020 | Japan | | Circulatory Risk in Communities Study, free of stroke | NT-proBNP | 4393/35 | 60.8/41 | -/5 | Medical records | 4.7 years | 7 |
| Edwards,  2020 | Canada | | ≥65 yrs, free of AF | LA diameter | 19265/196 | 70.9/46 | 70/30 | ICD-10 codes | 5 years | 8 |
| Folsom,  2013 | USA | | Atherosclerosis Risk in Communities study, 54 to 73 yrs, free of stroke | NT-proBNP | 10902/444 | 62.8/44 | -/20 | Combination of  self-reports and  ICD-9 codes | 11.3 years | 8 |
| Hirota,  2021 | Japan | | Shinken Database, free of oral anticoagulation treatment | LA diameter | 18511/116 | 58.6/61 | 39/17 | Self-report | 824.8 days | 5 |
| Kamel,  2014 | USA | | Multi-Ethnic Study of Atherosclerosis cohort, 45-84 yrs free of cerebrovascular or cardiovascular disease | PTFV1,  P-wave area,P-wave  duration | 6741/121 | 62.1/53 | 45/13 | Combinations of clinical symptoms and brain  imaging | 8.5 years | 7 |
| Kamel,  2018 | USA | | Cardiovascular Health Study, ≥65 yrs without stroke and AF | PTFV1, left atrial  dimension, NT-proBNP | 3723/585 | 73/40 | -/15 | Combinations of clinical symptoms and brain  imaging | 12.9 years | 9 |
| Kamel,  2015 | USA | | Atherosclerosis Risk in Communities study, 45 to 64 yrs, free of AF | PTFV1 | 14542/904 | 54/55 | -/11 | Medical records | 22 years | 7 |
| Kamel,  2021 | USA | | Cardiovascular Health Study, ≥65 yrs free of stroke and AF | LA reservoir strain | 4000/651 | 73/42 | -/14 | Combinations of clinical symptomsand brain  imaging | 12.9 years | 8 |
| Karas,  2012 | USA-+ | | Strong Heart Study, 45-74 yrs, free of cardiovascular disease | LA diameter | 2391/138 | 59.2/35 | -/45 | ICD-9 codes | 12 years | 7 |
| Kurl,  2009 | Finland | | Kuopio Ischemic Heart Disease Risk Factor study, free of stroke and AF | NT-proBNP | 905/31 | 56.1/100 | 33/6 | ICD-9 and  ICD-10 codes | 9.6 years | 7 |
| Leung,  2018 | The  Netherlands | | Patients with first  diagnosis of AF | LA reservoir strain,  P-wave to A’ duration on tissue  Doppler  imaging | 1361/100 | 65.2/74 | 70/14 | Medical records | 7.9 years | 6 |
| Li,  2021 | China | | Northeast China RuralCardiovascular Health Study, ≥35 yrs | LA diameter, LA diameter indexed by body surface area | 10041/261 | 53.8/48 | 50/10 | Medical records | 4.66 years | 7 |
| Liao,  2020 | China | | AF patients without valvular heart diseases | LA strain,  LA strain  rate | 1457/111 | 71.6/56 | 64/38 | Medical records | 37.6  months | 6 |
| Lind,  2022 | Sweden | | Prospective  Investigation of the  Vasculature in UppsalaSeniors, 70 yrs | NT-proBNP | 1016/89 | 70/50 | -/14 | ICD-8, ICD-9  and ICD-10  codes | 15 years | 7 |
| Mosquera,  2011 | Spain | | ≥18 yrs | LA diameter | 52639/2314 | 61.8/52.9 | 48/25 | ICD-9 codes | 5.5 years | 8 |
| Okin,  2016 | USA | | Losartan Intervention For Endpoint Reduction study, 55 to 60 yrs hypertensive patients, free of AF | PTFV1 | 1879/45 | 57.3/53 | 100/12 | Medical records | 4.8 years | 6 |
| O'Neal,  2016 | USA | | Atherosclerosis Risk  in Communities study,45 to 64 yrs, free of stroke and AF | Interatrial  block | 14716/916 | 54/45 | -/11 | Self-reports and medical records | 22 years | 8 |
| Portegies,  2015 | The  Netherlands | | Rotterdam Study, ≥55 yrs, free of stroke and TIA | NT-proBNP | 5611/425 | 67.9/42 | -/11 | Medical records | 9.3 years | 7 |
| Rutten,  2009 | The  Netherlands | | Rotterdam study, ≥55 yrs free of stroke | NT-proBNP | 5063/124 | 68/40 | 60/9 | ICD-10 codes | 5 years | 7 |
| Soliman,  2009 | USA | | Atherosclerosis Risk  in Communities study,45 to 64 yrs, free of AF | PTFV1, P-wave duration, P-wave area | 15429/599 | 54.2/55 | 35/12 | Self-reports and medical records | 6.97 years | 8 |
| Wang,  2022 | China | | ≥65 yrs with paroxysmal non-valvular atrialfibrillation | Interatrial  block  (maximum P-wave  duration) | 516/31 | 85.53/95 | 76/39 | Combinations of clinical symptomsand brain  imaging | 19 months | 4 |
|  | | TIA, transient ischemic attack; AF, atrial fibrillation; LA, left atrial; NT-proBNP, N-terminal pro-brain natriuretic peptide; ICD, International Classification of Diseases; PTFV1, P-terminal force in the precordial lead V1. | | | | | | | | |

| **Supplementary Table 4.** Quality assessment of included studies | | | | | | | | | |
| --- | --- | --- | --- | --- | --- | --- | --- | --- | --- |
| Study | Selection |  |  |  | Comparability | Outcome |  |  | score |
|  | Represent-ativeness of the  cases | Selection  of the non-exposure  cohort | Ascertainment  of exposure | Demonstration that outcome of interest wasnot present at start of study | Comparability  of cohorts on  the basis of the design or  analysis | Assessment  of outcome | Was follow-up long enough  for outcome to occur | Adequacy of follow-  up of  cohorts |  |
| Barnes, 2004 | ☆ | ☆ | ☆ |  | ☆ | ☆ | ☆ |  | 6 |
| Broughton, 2016 | ☆ | ☆ | ☆ | ☆ | ☆ | ☆ | ☆ |  | 7 |
| Castelnuovo, 2019 | ☆ | ☆ | ☆ |  | ☆☆ | ☆ | ☆ |  | 7 |
| Doi, 2011 | ☆ | ☆ | ☆ | ☆ | ☆ | ☆ |  | ☆ | 7 |
| Ebihara, 2020 | ☆ | ☆ | ☆ | ☆ | ☆☆ | ☆ |  |  | 7 |
| Edwards, 2020 | ☆ | ☆ | ☆ | ☆ | ☆☆ | ☆ |  | ☆ | 8 |
| Folsom, 2013 | ☆ | ☆ | ☆ | ☆ | ☆☆ | ☆ | ☆ |  | 8 |
| Hirota, 2021 | ☆ | ☆ | ☆ | ☆ | ☆ |  |  |  | 5 |
| Kamel, 2014 | ☆ | ☆ | ☆ | ☆ | ☆ | ☆ | ☆ |  | 7 |
| Kamel, 2018 | ☆ | ☆ | ☆ | ☆ | ☆☆ | ☆ | ☆ | ☆ | 9 |
| Kamel, 2015 | ☆ | ☆ | ☆ | ☆ | ☆☆ |  | ☆ |  | 7 |
| Kamel, 2021 | ☆ | ☆ | ☆ | ☆ | ☆☆ | ☆ | ☆ |  | 8 |
| Karas, 2011 | ☆ | ☆ | ☆ | ☆ | ☆ |  | ☆ |  | 6 |
| Kurl,2009 |  | ☆ | ☆ | ☆ | ☆ | ☆ | ☆ | ☆ | 7 |
| Leung, 2018 |  | ☆ | ☆ |  | ☆ | ☆ | ☆ | ☆ | 6 |
| Li, 2021 | ☆ | ☆ | ☆ | ☆ | ☆ | ☆ |  | ☆ | 7 |
| Liao, 2020 |  | ☆ | ☆ | ☆ | ☆ | ☆ |  | ☆ | 6 |
| Lind, 2022 | ☆ | ☆ | ☆ | ☆ | ☆ | ☆ | ☆ |  | 7 |
| Mosquera, 2011 | ☆ | ☆ | ☆ | ☆ | ☆ | ☆ | ☆ | ☆ | 8 |
| Okin, 2016 |  | ☆ | ☆ | ☆ | ☆☆ | ☆ |  |  | 6 |
| O'Neal, 2016 | ☆ | ☆ | ☆ | ☆ | ☆☆ | ☆ | ☆ |  | 8 |
| Portegies, 2015 | ☆ | ☆ | ☆ | ☆ | ☆ |  | ☆ | ☆ | 7 |
| Rutten, 2009 | ☆ | ☆ | ☆ | ☆ | ☆ | ☆ |  | ☆ | 7 |
| Soliman, 2009 | ☆ | ☆ | ☆ | ☆ | ☆ | ☆ | ☆ | ☆ | 8 |
| Wang, 2022 |  | ☆ | ☆ | ☆ | ☆ |  |  |  | 4 |

**Supplementary Figure 1.** Funnel plots for the effect estimates of electrophysiological markers in this meta-analysis. A, PTFV1 as a categorical variable. B, PTFV1 as a continuous variable. C, maximum P-wave duration. D, mean P-wave duration. E, advanced interatrial block. F, maximum P-wave area. G, mean P-wave area. PTFV1 indicates P-terminal force in the precordial lead V1.


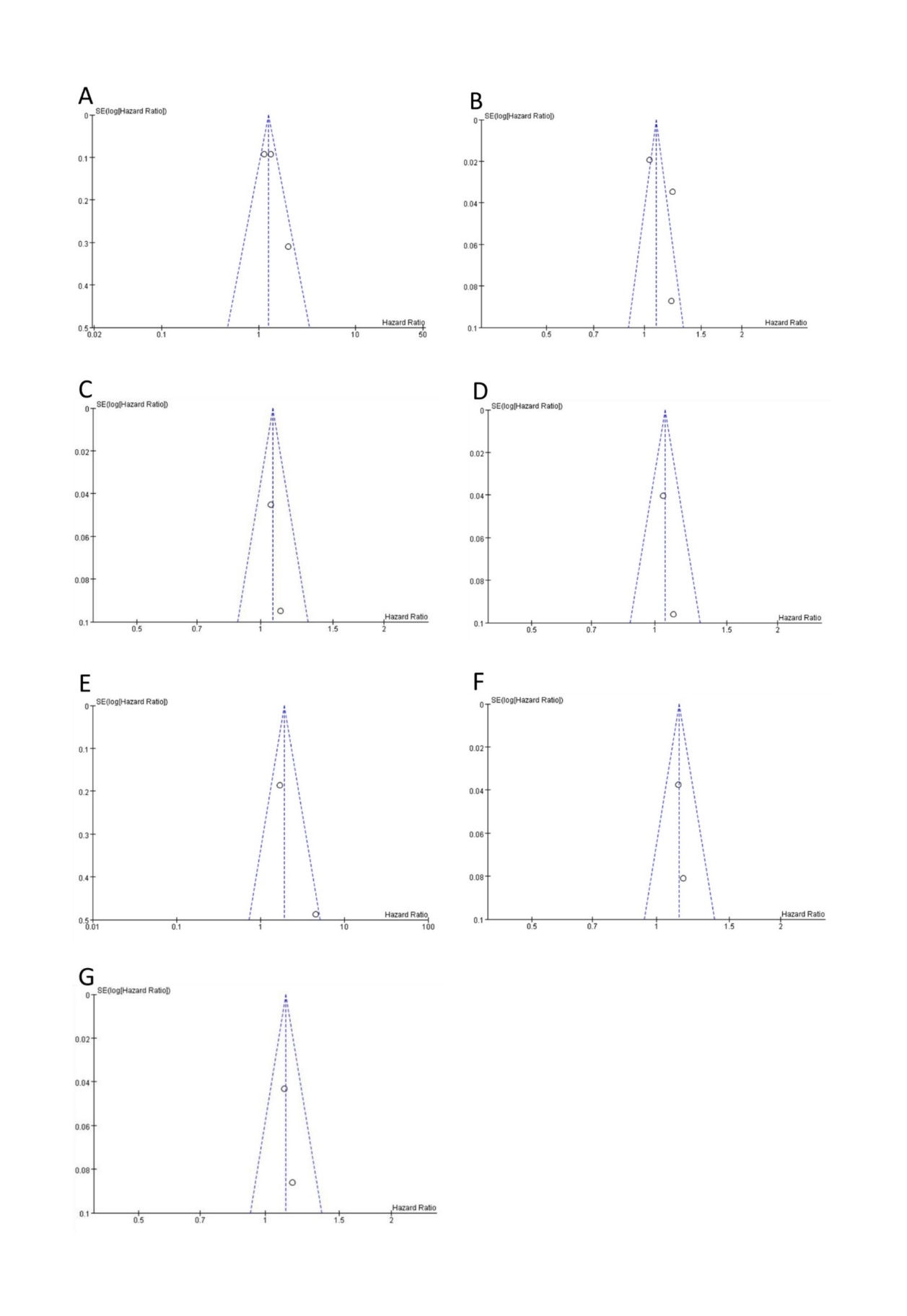


**Supplementary Figure 2.** Funnel plots for the effect estimates of structural and functional markers in this meta-analysis. A, LA diameter as a categorical variable. B, LA diameter as a continuous variable. C, LA reservoir strain. LA indicates left atrial.


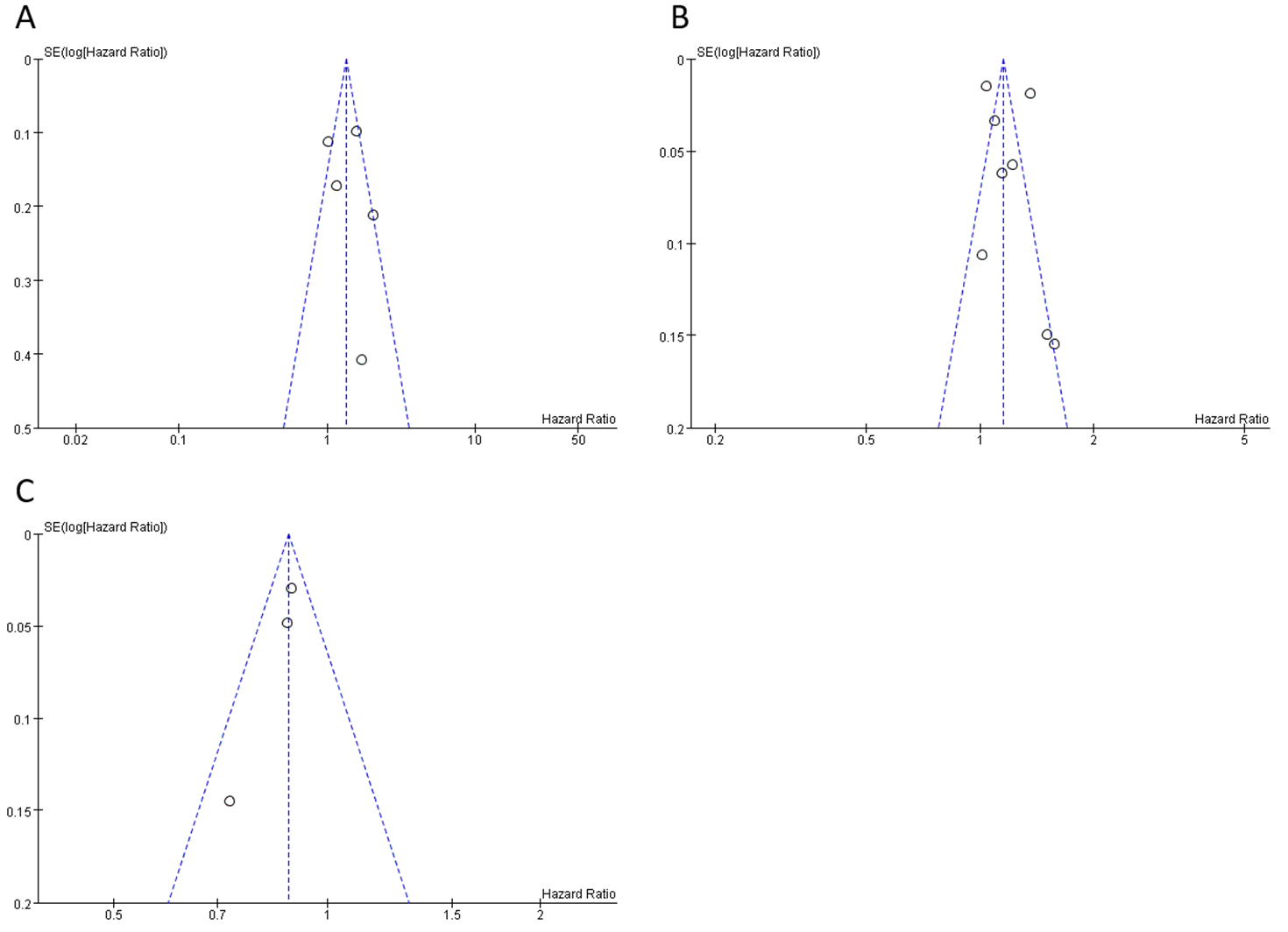


**Supplementary Figure 3.** Funnel plots for the effect estimates of NT-proBNP in this meta-analysis. A, NT-proBNP as a categorical variable. B, NT-proBNP as a continuous variable. C, sensitivity analysis of NT-proBNP as a categorical variable. D, sensitivity analysis of NT-proBNP as a continuous variable. NT-proBNP indicates N-Terminal Pro-Brain Natriuretic Peptide.


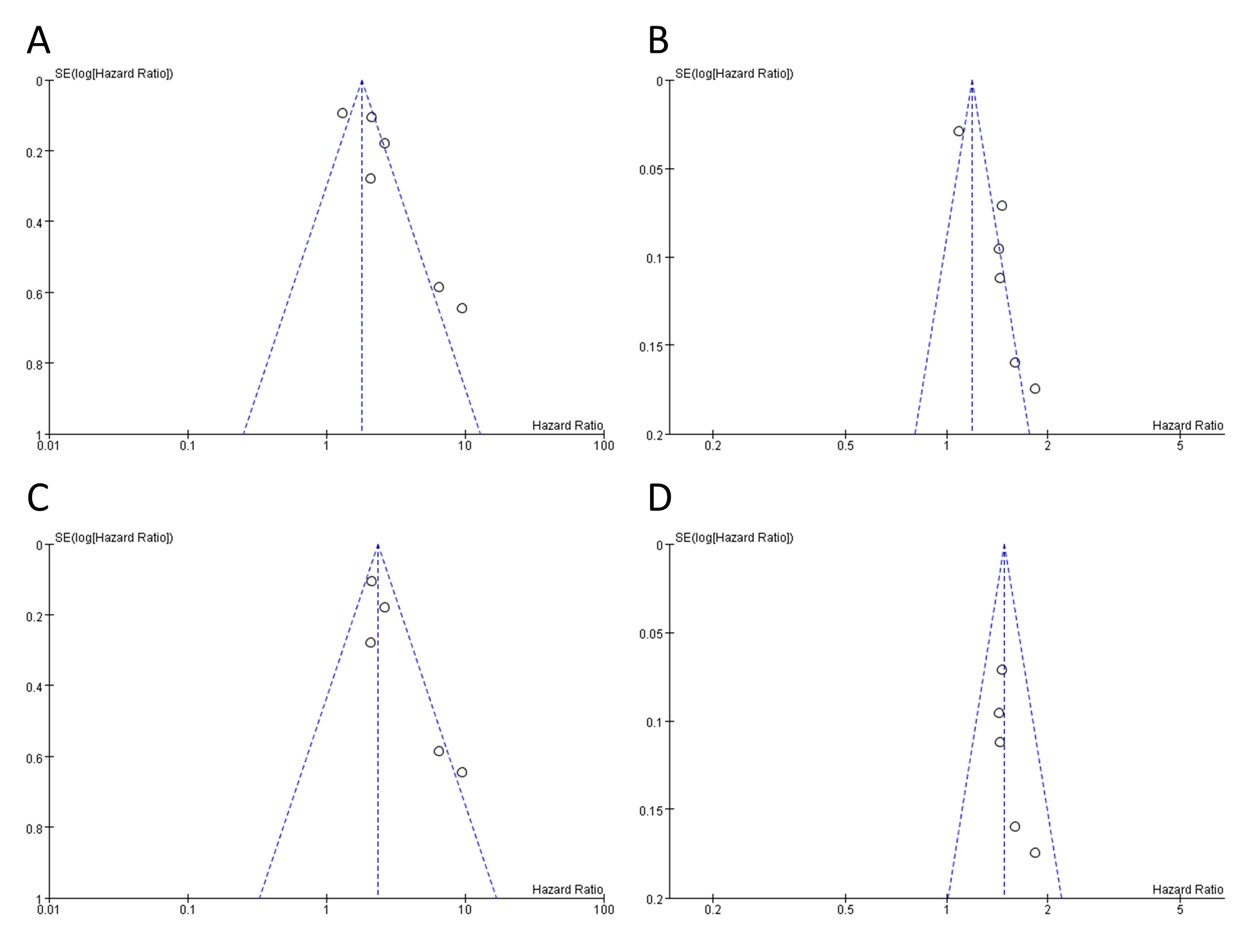

Supplement: Supplementary file 1 — Supplementary file1 (DOCX 332 KB) [file 415_2023_11693_MOESM1_ESM.docx]
